# Evaluation of Surgical Treatment of Oroantral Fistulae in Smokers Versus Non-Smokers

**DOI:** 10.3390/medicina56060310

**Published:** 2020-06-23

**Authors:** Adi Sella, Yehonatan Ben-Zvi, Leon Gillman, Gal Avishai, Gavriel Chaushu, Eli Rosenfeld

**Affiliations:** 1 Oral and Maxillofacial Surgery Unit, Shaare Zedek Medical Center, P.O.B 3235, 9103102 Jerusalem, Israel; 2 Department of Oral and Maxillofacial Surgery, Rabin Medical Center, Beilinson Campus, 49100 Petach-Tikva, Israel; yonident@gmail.com (Y.B.-Z.); gillman.leon@gmail.com (L.G.); drgalavishai@gmail.com (G.A.); gabi.chaushu@gmail.com (G.C.); eliros@gmail.com (E.R.)

**Keywords:** preprosthetic surgery, oroantral fistula (OAF), wound healing, smoking

## Abstract

*Background and Objectives*: Smoking has been found to interfere with wound healing processes. Therefore, the purpose of this study was to compare surgical treatment of oroantral fistulae (OAFs) in smokers and non-smokers. *Materials and Methods*: Medical records of all consecutive patients who underwent surgical closure of OAFs between 2003 and 2016 at the oral and maxillofacial surgery department, Rabin Medical Center, Israel were reviewed. Patients’ demographic data, preoperative signs and symptoms, surgical method of repair, and postoperative complications were recorded. *Results*: The cohort consisted of 38 smokers and 59 non-smokers. Age and gender distributions were similar in both groups. The main etiology in both groups was tooth extraction, followed by pre-prosthetic surgery in smokers and odontogenic infection in non-smokers (*p* = 0.02). Preoperative conditions were not significantly different between smokers and non-smokers in terms of size of soft tissue fistula and bony defect, chronic sinusitis and foreign bodies inside the sinus. OAFs were repaired by local soft tissue flaps without consideration of smoking status. Smokers experienced more moderate-severe postoperative pain (*p* = 0.05) and requested more weak opioids (*p* = 0.06). Postoperative complications included infection, delayed wound healing, residual OAF, pain, sensory disturbances and sino nasal symptoms. These were mostly minor and tended to be more frequent in smokers (*p* = 0.35). Successful closure of OAFs was obtained in all patients except one smoker who required revision surgery. *Conclusions*: Smokers may be more susceptible to OAFs secondary to preprosthetic surgery. In this cohort, there was no statistically significant difference in outcome between smokers and non-smokers in terms of failure. However, smokers tended to have more severe postoperative pain and discomfort and to experience more postoperative complications. Further studies with larger sample sizes should be conducted to validate these results.

## 1. Introduction

**Figure 1:**
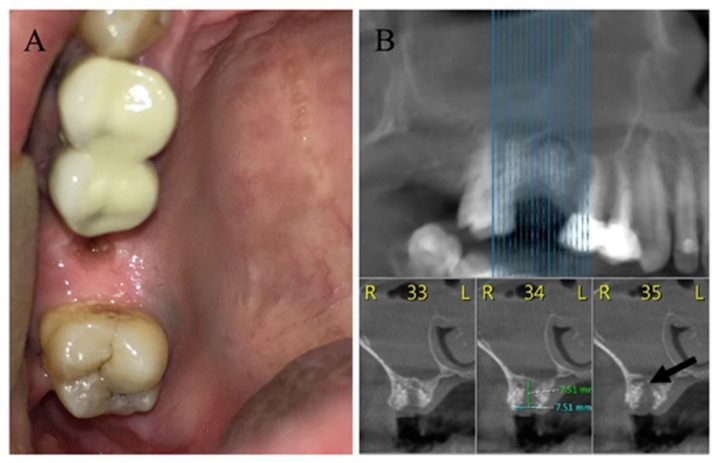
(**A**) Intraoral image of a 35-year-old smoker with an oroantral fistula (OAF) in the right posterior maxilla, secondary to removal of a failed dental implant and socket preservation. (**B**) Cone beam computed tomography (CBCT) of the patient presented in Figure 1A, demonstrating bony defect with discontinuity of the sinus floor (black arrow). The entire sinus is radio-opaque due to inflammation.

Oroantral fistulae (OAFs) are pathological tracts connecting the oral cavity and maxillary sinus. These communications are usually iatrogenic, secondary to surgical procedures in the posterior maxilla, such as tooth extraction, sinus augmentation and dental implants (Figure 1), but may also result from pathology or trauma [1,2,3].

Small oroantral communications (OACs), up to 3 mm, tend to heal spontaneously by secondary healing. However, larger communications (>3 mm), and those accompanied by inflammation, usually require surgical closure to prevent epithelialization and formation of chronic OAFs [1,3,4,5,6,7,8]. OACs make the maxillary sinus susceptible to retrograde contamination by oral bacteria and the subsequent development of odontogenic sinusitis. Common symptoms of OAFs include fluid passage into the nasal cavity during meals, purulent discharge, postnasal drip (PND), nasal congestion, pain and halitosis [1,3,9].

Early surgical closure is advisable, as approximately 50% of patients will develop sinusitis after 48 h and up to 90% after two weeks [1,9,10,11]. In a study by Puglisi et al., odontogenic sinusitis was always iatrogenic in nature, with sinus lift procedures as the leading etiology followed by tooth extraction. In their study, odontogenic sinusitis was considered as a polymicrobial infection. The predominant aerobes were Staphylococcus aureus and Streptococcus pneumoniae, while the more frequent anaerobes were Peptostreptococcus species and Prevotella species [12].

Various soft tissue flaps have been described in the literature for closure of OAFs. Local flaps include the buccal advancement flap, palatal rotation flap and the pedicled buccal fat pad flap. Distant soft tissue flaps include the tongue flap and temporalis fascia flap. Each flap has its advantages and disadvantages. Alternative methods which use bone grafts and alloplastic materials to fill the bony defect have also been reported. Several aspects should be considered when selecting the surgical approach to close OAFs—size, location, sinus disease, condition of tissues available for repair, dental status, possible placement of dental implants in the future and surgeon’s preferences [1,3,5,6,7,13,14].

The mechanisms by which smoking affects wound healing are not completely understood. One possible explanation is that substances in tobacco and its smoke, particularly nicotine, cotinine, carbon monoxide, and hydrogen cyanide are cytotoxic to the cells involved in wound healing. Nicotine increases platelet adhesiveness, raising the risk of microvascular occlusion and tissue ischemia [15,16]. Carbon monoxide along with other chemicals produced during the combustion of tobacco lead to the reduction in capillary blood flow. A single cigarette can reduce the peripheral blood velocity by 40% in one hour [17].

Smoking seems to impair wound healing in plastic and orthopedic surgeries. The literature suggests several mechanisms by which smoking interferes with wound healing processes including inadequate perfusion and tissue ischemia, poor neovascularization, reduced proliferation of red blood cells, macrophages and fibroblasts, impaired leukocyte chemotaxis and phagocytosis, impaired osteoblastic activity and disruption of bone remodeling [18,19,20]. On the other hand, the effect of smoking on outcome of functional endoscopic sinus surgery (FESS) is inconsistent, even though it is well accepted that smoking impairs ciliary function and contributes to development of chronic sinusitis [21,22]. 

To date, the effect of smoking on the surgical treatment of OAFs has not been reported. The aim of the present study was to compare the outcome of surgical treatment of OAFs in smokers and non-smokers.

## 2. Materials and Methods

This retrospective study protocol was approved by the institutional ethics committee of Rabin Medical Center (No. 0789-16-RMC). Medical records of all consecutive patients who underwent surgical closure of OAFs between 2003 and 2016 at the department of oral and maxillofacial surgery, Rabin Medical Center, Beilinson Campus, Petah Tikva, Israel were reviewed. 

Exclusion criteria were as follows:

1. OAFs secondary to excision of pathology, other than odontogenic cyst or granuloma
2. OAFs s/p sequestrectomy in patients with Medication Related Osteonecrosis of the Jaws (MRONJ)
3. History of radiation therapy to the maxilla
4. Cases with insufficient data, or no follow-up visit after surgery
5. Former smokers (10 patients) were excluded due to insufficient data regarding smoking cessation period [18].

A structured form was used to collect the following data:

1. Smoking status—non-smokers vs. smokers. Non-smokers were defined as those who had never smoked, whereas, smokers were defined as those who reported being current smokers [18,19,23].
2. Age
3. Gender
4. Medical status based on the American Society of Anesthesiologists (ASA) physical status classification [24]
5. OAFs etiology—extraction, odontogenic infection, pathology, preprosthetic surgery (insertion of dental implants and sinus augmentation)
6. OAFs size—measured clinically, in millimeters, as maximum diameter of soft tissue fistula
7. Size of bony defect underlying OAFs—measured in millimeters as the maximum diameter of bony defect, either clinically during surgery or radiographically on a preoperative computed tomography (CT) scan, Cone beam computed tomography (CBCT), panoramic or Water’s view. Whenever a CT was used the measurement was conducted on the coronal reconstruction, and when a CBCT was used the panoramic reconstruction was used.
8. Soft tissue fistula surface area (soft tissue deficit)—calculated as π*(0.5*soft tissue fistula diameter) ^2^.
9. Bone defect surface area—calculated as π*(0.5*bony defect diameter) ^2^
10. Soft tissue deficit relative to underlying bone defect—calculated as the ratio between the soft tissue fistula surface area relative to the bone defect surface area.
11. Maxillary sinusitis—was diagnosed when presenting symptoms of purulent nasal discharge accompanied by nasal obstruction, facial pain (pressure, fullness, or both), with radiographic signs of inflamed paranasal sinuses or signs of purulent mucus or polyps on medical examination [25,26].
12. History of previous FESS.
13. Preoperative radiographic appearance of the antral cavity was determined based on either a CT scan, CBCT, panoramic view, or water’s view, and categorized into clear, thickened mucosal lining (>2 mm) or occluded sinus. Presence and type of foreign bodies inside the antral cavity were also recorded [25].
14. Operative time in minutes.
15. Type of flap used for fistula repair—Palatal flap, buccal advancement flap, buccal fat pad, or combinations.
16. Caldwell-Luc operation (yes/no), either with or without inferior meatal antrostomy.
17. Postoperative follow up time (months).
18. Duration of hospitalization (days).
19. Analgesic consumption during hospitalization (mean analgesic dose/day).
20. Postoperative pain level during hospitalization was categorized into no pain, mild, moderate, and severe pain based on the type of analgesics consumed and according to the world health organization (WHO) analgesic ladder [27]
21. Postoperative complications included:
  a. Bleeding
  b. Infection of surgical site
  c. Postoperative pain > four weeks
  d. Delayed wound healing—defined as incomplete soft tissue healing of the flap or incomplete soft tissue coverage of the denuded palate observed eight weeks postoperatively [3].
  e. Infraorbital sensory disturbance (paresthesia/hypoesthesia) lasting longer than eight weeks postoperatively [28]
  f. Epiphora
  g. Persistent sino nasal symptoms of chronic rhinitis, nasal congestion, or sinusitis.
  h. Residual OACs—characterized by symptoms of air/fluid escape through surgical site. Follow up was continued until spontaneous closure was obtained [3,28,29].
22. Failure was defined as residual or recurrent OAF observed 12 weeks postoperatively, requiring further surgical intervention [3].

### 2.1. Surgical Procedure

Patients with acute maxillary sinusitis were treated preoperatively with systemic antibiotics combined with antral irrigations with chlorhexidine gluconate 0.2% through the fistula. Surgical closure of OAFs was performed under general anesthesia with nasotracheal intubation in all patients. Surgery began with fistulectomy, after which a mucoperiosteal flap was developed to expose the bony defect. Debridement and irrigation of the antral cavity were performed through the existing communication or by a Caldwell-Luc procedure as needed. In cases of the Caldwell-Luc operation, an inferior meatal antrostomy was performed at the discretion of the senior surgeon, along with packing using a Foley catheter. OAFs were repaired using one or more of the following soft tissue flaps—buccal advancement flap, palatal rotation flap and pedicled buccal fat pad flap. The criteria for flap selection included size and location of soft tissue fistula, size of underlying bony defect, condition of soft tissues available for repair, dental status, and surgeon’s preferences. Closure was obtained using resorbable sutures. All patients received perioperative intravenous antibiotics (amoxicillin clavulanate1g or clindamycin 600 mg) and 20 mg of Dexacort.

### 2.2. Postoperative Care

All patients received intravenous antibiotics (amoxicillin clavulanate 1 g TID or clindamycin 600 mg TID) while admitted, followed by oral antibiotics, for up to one week, upon discharge. Chlorhexidine gluconate 0.2% mouth rinses were administered routinely for two weeks. Soft and cold diet restrictions were advised during the first 24 h postoperatively. Patients were also advised to avoid nose blowing two weeks postoperatively. Nasal drops containing phenylephrine were prescribed for the first three days postoperatively, followed by sodium chloride 0.9% drops as needed to reduce nasal congestion. Analgesics were administered upon demand, in a stepped approach, based on visual analog scale (VAS). First line agents were non-opioids, such as Dipyrone 1 gr, Paracetamol 1 gr, IM Diclofenac 75 mg, or combinations of the above. In cases of severe pain, weak opioids (such as IV Tramal 100 mg) and combination regimens with non-opioids were used. 

### 2.3. Statistical Analysis

Statistical analysis was generated using SAS Software, Version 9.4. Continuous variables were presented by mean ± standard deviation (SD), whereas categorical variables were presented by number (n) and percentage. Student’s *t*-test was used to compare the value of continuous variables between smokers and non-smokers. Fisher’s exact test was used to compare the value of categorical variables between these study groups. Two-sided *p*-values less than 0.05 were considered statistically significant.

## 3. Results

Medical records of 97 patients were included. The cohort consisted of 38 (39.2%) smokers and 59 (60.8%) non-smokers. Demographic data are presented in Table 1. The mean age was 51.3 ± 12.0 years in smokers and 50.4 ± 16.0 years in non-smokers (*p* = 0.75). In both groups, the male-to-female ratio was in favor of males. This ratio was higher in smokers compared to non-smokers (2.2:1 vs. 1.2:1, *p* = 0.2). Medical status, as measured by ASA classification (Table 1), was significantly better among non-smokers (ASA 1,2,3 was 33.9%, 59.3%, 6.8% in non-smokers vs. 0%, 89.5%, and 10.5% in smokers respectively, *p* = 0.001). 

Smokers and non-smokers differed significantly (*p* = 0.02) in the main etiologies of OAFs formation (Table 2). In both groups, the leading cause of OAFs formation was tooth extraction. This was followed by preprosthetic surgery in smokers (36.8%) as opposed to odontogenic infection in non-smokers (23.7%). OAFs secondary to preprosthetic surgery were three times more common in smokers compared to non-smokers (36.8% vs. 11.9%). 

The preoperative conditions of the two study groups are presented in Table 3. In this cohort, the average soft tissue fistula diameter was insignificantly greater in smokers compared to non-smokers (5.7 ± 4.2 vs. 4.3 ± 3.2, *p* = 0.13). However, the mean diameters of the underlying bone defects were similar in these two groups (13.9 ± 9.7 mm vs. 14.0 ± 9.9 mm). The calculated ratio between the soft tissue deficit (surface area, mm^2^) relative to the underlying bony defect (surface area, mm^2^) was also greater in smokers compared to non-smokers (1.5 ± 5.3 vs. 0.4 ± 0.6). This tendency was not statistically significant (*p* = 0.35). 

In both groups OAFs were often associated with signs and symptoms of maxillary sinusitis. Clinical symptoms of sinusitis were reported in 84.2% of smokers and 79.7% of non-smokers (*p* = 0.79). Of those, 15.6% of smokers and 8.5% of non-smokers were symptomatic despite previous FESS surgery. Similarly, preoperative radiographic features of sinus pathology were observed in 92.1% of smokers and 83.0% of non-smokers (*p* = 0.35). In this cohort, we also found that smokers presented with foreign bodies inside the antral cavity nearly twice as often as non-smokers (21.0% vs. 11.9%, *p* = 0.13). These included, in descending order, bone graft material, tooth roots, dental implants and other dental materials. 

**Figure 2:**
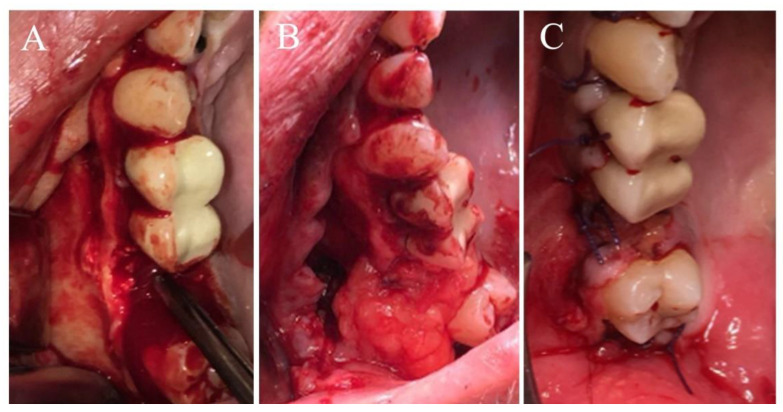
(**A**) Intraoral image taken during surgical closure of OAF in the right posterior maxilla—buccal flap raised; metal instrument inserted into the bony defect. (**B**) Bony defect covered with buccal fat pad. (**C**) Immediate postoperative intraoral image showing two layered closure of the OAF using buccal fat pad (inner layer) and a buccal advancement flap (outer layer).

Surgical repair of 97 OAFs were evaluated retrospectively. Operative data are presented in Table 4. In this cohort, OAFs were repaired by either palatal flap (*n* = 48, 49.5%), buccal advancement flap (*n* = 13, 13.4%), buccal fat pad combined with a buccal flap (*n* = 29, 29.9%, Figure 2) or a combination of all three flaps (*n* = 7, 7.2%). Flap distribution followed the same trend in smokers and non-smokers (*p* = 0.71, Table 4). A Caldwell-Luc procedure was performed in addition to fistula repair in 23 (60.5%) smokers and 48 (81.3%) non-smokers with evidence of sinus pathology. Overall, the mean operative time was not significantly different between smokers and non-smokers (76.4 ± 26 vs. 74.6 ± 25 min, *p* = 0.74). 

Postoperative data are presented in Table 5. The mean follow-up period was not significantly different between the groups (8.5 ± 12.8 vs. 7.3 ± 11.6 months, *p* = 0.65). Hospitalization period was 3.6 ± 1.7 days in smokers and 4.0 ± 1.9 days in non-smokers (*p* = 0.34). Most patients, regardless of smoking status, had no or only mild postoperative pain during hospital stay. However, smokers experienced more moderate-severe postoperative pain (23.7% vs. 11.9%, *p* = 0.05), and required more weak opioids, such as Tramal, compared to non-smokers (0.6 vs. 0.1, Mean Dose, *p* = 0.06). 

**Figure 3:**
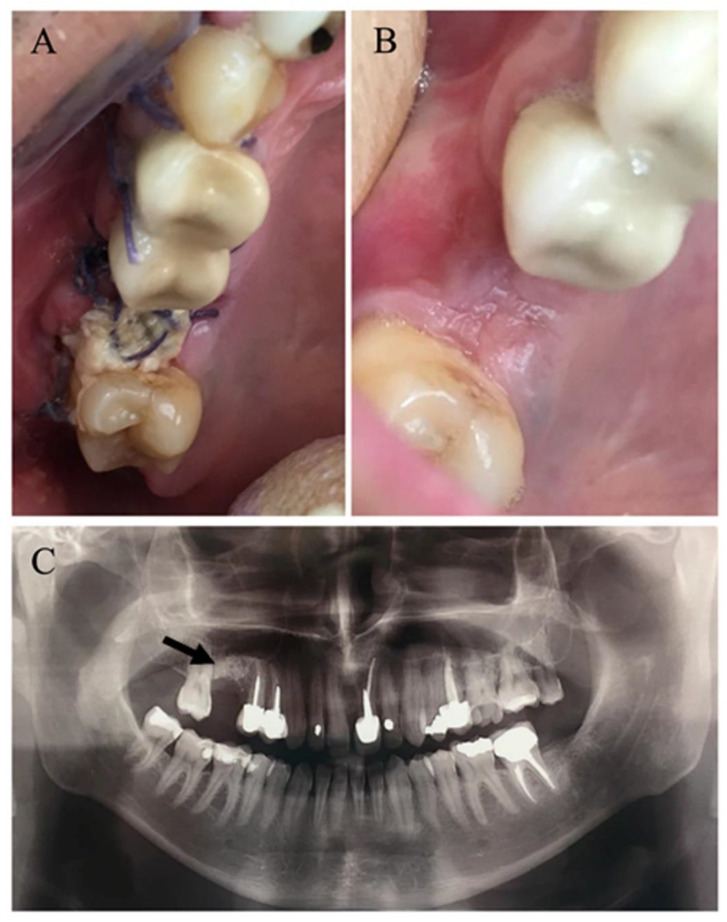
Postoperative course of the patient presented in Figure 1 and Figure 2. (**A**) Intraoral image taken one week postoperatively, showing some granulation tissue and fibrin in surgical site. (**B**) Intraoral image taken one month postoperatively, showing complete epithelialization with no residual fistula. (**C**) Panoramic view taken three months postoperatively, presenting complete consolidation (arrow) of the previously area of OAF.

In this cohort, successful closure of OAF was obtained in all patients except one, who required a second surgical intervention, yielding a success rate of 98.9% (Figure 3). Uneventful healing was noted in most patients of both groups (65.8% smokers vs. 76.3% non-smokers). Postoperative complications (Table 6) were mostly minor, and although not statistically significant, were somewhat more frequent in smokers (34.2% vs. 23.7%, *p* = 0.35). Two non-smoking patients (3.4%) had surgical site bleeding in the immediate postoperative period. Both were sutured under local anesthesia and healed uneventfully. Surgical site infection was observed in four smokers vs. two non-smokers (10.5% vs. 3.4%, *p* = 0.2). They were treated medically with complete resolution. Persistent postoperative pain, lasting longer than four weeks was reported by five smokers vs. three non-smokers (13.2% vs. 5.1%, *p* = 0.25) who were treated by oral analgesics. Epiphora was noted in one non-smoking patient (1/59, 1.7%) and resolved spontaneously within eight weeks. 

During the early postoperative period, residual OACs were observed in 10 patients (6 smokers vs. four non-smokers, *p* = 0.18), who were treated conservatively by antiseptic irrigations and monitored closely. In nine patients, the OACs resolved spontaneously by secondary healing up to eight weeks postoperatively. However, one smoker with a persistent OAF 12 weeks postoperatively, was defined as failure (1/97, 1.03%) and required revision surgery. Delayed soft tissue healing, eight weeks postoperatively, was noted in just two smokers (5.26%, *p* = 0.15)—at the denuded palate (*n* = 1) and the buccal flap (*n* = 1). One was treated by debridement and the other with antiseptic mouthwash with complete healing.

One smoker and four non-smokers (2.6% vs. 6.8%, *p* = 0.64) had persistent symptoms of chronic rhinitis, nasal congestion, or sinusitis, later than eight weeks postoperatively, with no evidence of OAF. They were referred to an otolaryngologist for further evaluation and treatment. Infraorbital sensory disturbances presenting as hypoesthesia or paresthesia, lasting longer than eight weeks postoperatively, were noted in three smokers (7.9%, *p* = 0.06). This most probably resulted from pressure or traction injury to the infraorbital nerve during surgery. All four patients remained under follow up for observation. 

## 4. Discussion 

Medical records of 97 patients who underwent surgical repair of OAF were reviewed. The cohort consisted of 38 smokers and 59 non-smokers. In both groups, age and gender distributions were similar, with a male-to-female ratio in favor of males, and a mean age of 50.8 years. However, non-smokers were generally healthier than smokers, as reflected by the ASA classification (*p* = 0.001, Table 1). 

The distribution of etiologies of OAF formation was significantly different between smokers and non-smokers (*p* = 0.02). The main etiology in both groups was tooth extraction followed by pre-prosthetic surgery in smokers compared with odontogenic infection in non-smokers. These differences could be explained by the perception that smoking enhances periodontal disease progression and diminishes response to therapy [30]. Therefore, smokers tend to be more susceptible to early edentulism and to seek prosthodontic solutions. Moreover, smoking seems to be a risk factor for dental implant failure, postoperative infection and marginal bone loss [23,31,32,33]. In this cohort, we also found higher incidence of foreign bodies (implants, bone graft materials and tooth roots) inside the antral cavity in smokers (21.0% vs. 11.9%, *p* = 0.13). This trend, although not statistically significant, is in accordance with our observation that OAFs as complications of pre-prosthetic surgery were three times more frequent in smokers compared to non-smokers (Table 2). 

Recent studies by Isola et al. suggest that abnormal serum vitamin D levels as well as high serum glycosylated hemoglobin (HbA1c) levels have adverse effects on periodontal health leading to decreased clinical attachment level and tooth loss [34,35]. Tooth extraction and preprosthetic surgery secondary to periodontitis were not directly addressed in our study. It would be interesting to investigate these etiologies with respect to vitamin D and HbA1C levels in the formation of OAF in future studies.

In this cohort, the preoperative conditions were not significantly different between smokers and non-smokers in terms of size of soft tissue fistula relative to the bone defect, presence of sinus pathology and incidence of foreign bodies inside the sinus (Table 3). In both groups, most OAFs were associated with signs and symptoms of maxillary sinusitis (clinically and on imaging), which could be attributed to the long-standing fistulae treated in this cohort, leading to retrograde infection of the antral cavity by odontogenic bacteria. 

Although smoking has a negative effect on ciliary function, leading to mucostasis and sinus inflammation, and is known to interfere with wound healing processes [21,22,25], we did not find statistically significant differences in the preoperative conditions between the two groups in this cohort. Perhaps a larger sample size would have allowed for a statistical difference between the groups. 

In this cohort, all OAFs were surgically repaired using local soft tissue flaps—buccal advancement flap, palatal rotation flap, pedicled buccal fat pad flap or combinations. The Pedicled buccal fat pad flap combined with buccal advancement flap and or palatal rotation flaps were used to treat large communications (≥ 5mm), as described in the literature [5]. The treatment was primarily determined by size and location of the soft tissue fistula and underlying bony defect. The condition of soft tissues available for repair (scaring from previous surgery, remaining attached mucosa), dental status and surgeon’s preferences were also considered [5]. However, smoking status was not included in the operative considerations. The type of flap used for repair followed the same distribution trend in the two study groups (Table 4). 

In this cohort, the successful closure of OAF was obtained in all patients except one, who required a second surgical intervention, yielding a success rate of 98.9%. Uneventful healing was noted in most patients of both groups (65.8% smokers vs. 76.3% non-smokers). However, smokers experienced more moderate-severe postoperative pain during hospital stay (*p* = 0.05) and requested more weak opiates compared to non-smokers (*p* = 0.06). 

Postoperative complications included infection, delayed wound healing, residual OAC, pain, sensory disturbances and sinonasal symptoms. These were mostly minor, and although not statistically significant, were also more frequent in smokers (34.2% vs. 23.7%, *p* = 0.35). Residual OACs were not found to be associated with type of flap used for repair (*p* = 0.36, Fisher exact), and of the 10 residual OAC (10.3%), nine resolved spontaneously up to eight weeks postoperatively. Anavi et al. [3] reported lower incidence (7.9%) of residual OAC in their study of palatal flaps, which also healed spontaneously in the same time frame. Smoking status was not addressed in their work. Delayed soft tissue healing (>8 weeks postoperatively) was noted in two smokers at the denuded palate (*n* = 1) and the buccal flap used for fistula repair (*n* = 1). Although smoking status was not addressed, this complication was not reported in previous publications [3,10,14,36,37]. 

All these complications could be attributed to the adverse effects of smoking on wound healing, causing vasoconstriction induced hypoxia, attenuated inflammatory and proliferative responses and impeded collagen production [15,16,17,18,20]. Taking into account that smokers also presented a non-significant trend to have larger soft tissue fistulae—and that in order to achieve good repair, flap margins should be supported by intact underlying bone without tension [3,13]—it seems that smokers should anticipate less favorable surgical outcomes. 

However, of the 97 fistulas treated, we observed only one failure (1.03%, *p* = 0.39), which is lower compared to previous publications [3,10,29]. This failure could not be attributed to smoking alone, as there were confounding factors such diabetes mellitus, large fistula and bony defect, and an occluded osteomeatal complex. A review by Gazal G, regarding the effect of blood glucose levels on healing processes, emphasizes that patients with uncontrolled diabetes are at high risk of infection due to the high ketone levels in their blood [38]. This patient subsequently underwent FESS surgery and additional closure of OAF by a combination of palatal, buccal and buccal fat pad flaps with complete closure up to six months follow-up postoperatively. 

In summary, in the present cohort, despite the negative effect smoking has on wound healing processes, we did not find significant differences between smokers and non-smokers in surgical outcome with regards to failure. 

## 5. Conclusions

The results of this study imply that smokers may be more susceptible to OAFs secondary to preprosthetic surgery. In this cohort, we found no statistically significant difference in outcome between smokers and non-smokers with regards to failure. However, smokers tended to have more severe postoperative pain and discomfort and to experience more postoperative complications. Further studies with larger sample sizes should be conducted to validate these results. 

## Figures and Tables

**Table 1:** Demographic characteristics of study groups.

Variables	Non-Smokers	Smokers	*p*-Value
**No. of patients**	59	38	
**Gender**			
Male	32 (54.2%)	26 (68.4%)	0.2
Female	27 (45.8%)	12 (31.6%)	
**Age** (years, mean ± SD)	50.4 ± 16.0	51.3 ± 12.0	0.75
**ASA**			
I	20 (33.9%)	0	
II	35 (59.3%)	34 (89.5%)	0.001
III	4 (6.8%)	4 (10.5%)	

ASA: American Society of Anesthesiologists; SD: standard deviation.

**Table 2:** Etiology of OAF *.

Etiology	Non-Smokers	Smokers
**Non-elective surgery**		
Tooth extraction	36 (61.0%)	18 (47.4%)
Odontogenic infection	14 (23.7%)	6 (15.8%)
Pathology	2 (3.4%)	0
Total	52 (88.1%)	24 (62.5%)
**Elective Surgery**		
Preprosthetic surgery **	7 (11.9%)	14 (36.8%)

* Main etiologies of OAF formation in smokers and non-smokers (*p* = 0.02, fisher’s exact test) ** Preprosthetic surgery = sinus augmentation/dental implant insertion. OAF: oroantral fistula.

**Table 3:** Preoperative signs and symptoms.

Preoperative Signs and Symptoms	Non-Smokers	Smokers	*P* Value
OAF size			
Soft tissue fistula diameter (Mean ± SD, mm)	4.3 ± 3.2	5.7 ± 4.2	0.13
Bone defect diameter (Mean ± SD, mm)	13.9 ± 9.7	14.0 ± 9.9	0.97
Soft tissue deficit/underlying bony defect (Mean ± SD) *	0.4 ± 0.6	1.5 ± 5.3	0.35
Maxillary sinusitis—clinical symptoms	47 (79.7%)	32 (84.2%)	0.79
S/P FESS	4 (8.5%)	5 (15.6%)	0.3
Radiographic sinus pathology **	49 (83.0%)	35 (92.1%)	0.35
Foreign body inside the sinus	7 (11.9%)	8 (21.0%)	0.13
Implant	2 (3.4%)	1 (2.6%)	
Bone graft	3 (5.1%)	4 (10.5%)	
Tooth Root	0	3 (7.9%)	
Other	2 (3.4%)	0	

OAF = oroantral fistula, FESS = Functional endoscopic sinus surgery. *Soft tissue deficit = fistula’s surface area in mm^2^, Bony defect = bone defect surface area in mm^2^. **Radiographic sinus pathology = thickened mucosa/occluded sinus.

**Table 4:** Operative data.

Variable	Non-Smokers	Smokers	*p* Value
Operative time (Mean ± SD, Minutes)	76.4 ± 25.9	74.6 ± 25.2	0.74
Flap type			0.71
Palatal flap	32 (54.2%)	16 (42.1%)	
Buccal advancement flap	7 (11.9%)	6 (15.8%)	
Buccal fat pad + Buccal flap	16 (27.1%)	13 (34.2%)	
Buccal fat pad + Buccal flap + Palatal flap	4 (6.8%)	3 (7.9%)	
Caldwell-Luc operation	48 (81.4%)	23 (60.5%)	0.03

**Table 5:** Postoperative data.

Variable	Non-Smokers	Smokers	*p* Value
Follow up (Mean ± SD, Months)	7.3 ± 11.6	8.5 ± 12.8	0.65
Hospitalization period (Mean ± SD, Days)	4.0 ± 1.9	3.6 ± 1.7	0.34
Pain level during hospital stay *			0.05
None	17 (28.8%)	16 (48.5%)	
Mild	35 (72.9%)	13 (34.2%)	
Moderate	6 (10.2%)	5 (13.2%)	
Severe	1 (1.69%)	4 (10.53%)	
Tramal (Mean ± SD, Dose)	0.1 ± 0.36	0.6 ± 1.5	0.06

* Pain level during hospital stay—based on the type of analgesics consumed and according to the. WHO analgesic ladder [27]. Fisher’s exact test.

**Table 6:** Postoperative complications.

Postoperative Complications	Non-Smokers	Smokers	*p* Value
Bleeding	2 (3.4%)	0	0.52
Infection	2 (3.4%)	4 (10.5%)	0.2
Pain	3 (5.1%)	5 (13.2%)	0.26
Sensory disturbance	0	3 (7.9%)	0.06
Epiphora	1 (1.7%)	0	1
Delayed soft tissue healing	0	2 (5.3%)	0.15
Sinonasal symptoms	4 (6.8%)	1 (2.6%)	0.64
Residual OAC	4 (6.8%)	6 (15.8%)	0.18
Spontaneous closure of residual OAF	4 (100%)	5 (83.3%)	
**Failure**	0	1 (2.6%)	0.39

## References

[1] Dym H., Wolf J.C. (2012). Oroantral communication. Oral. Maxillofac. Surg. Clin. N. Am..

[2] Guven O. (1998). A clinical study on oroantral fistulae. J. Craniomaxillofac. Surg..

[3] Anavi Y., Gal G., Silfen R., Calderon S. (2003). Palatal rotation-advancement flap for delayed repair of oroantral fistula: A retrospective evaluation of 63 cases. Oral. Surg. Oral. Med. Oral. Pathol. Oral. Radiol. Endod..

[4] Fonseca R.J., Barber J.D., Matheson J.D. (2009). Oral and Maxillofacial Surgery.

[5] Park J.Y., Chun B.D., Hwang D.S. (2019). Versatility of the pedicled buccal fat pad flap of the management of oroantral fistula: A retrospective study of 25 cases. Maxillofac. Plast. Reconst. Surg..

[6] Visscher S.H., Van Minnen B., Bos R.R. (2010). Closure of oroantral communications: A review of the literature. J. Oral. Maxillofac. Surg..

[7] Von Wowern N. (1973). Correlation between the development of an oroantral fistula and the size of the corresponding bony defect. J. Oral. Surg..

[8] Von Wowern N. (1970). Frequency of oroantral fistulae after perforation of the maxillary sinus. Scand. J. Dent. Res..

[9] Bravo C.G., Minzer F.S., Fernández L. (2016). Odontogenic sinusitis, oro-antral fistula and surgical repair by Bichat’s fat pad: Literature review. Acta Otorrinolaringol. Esp..

[10] Yalcin S., Oncu B., Emes Y., Atalay B., Aktas I. (2011). Surgical treatment of oroantral fistulas: A clinical study of 23 cases. J. Oral. Maxillofac. Surg..

[11] Procacci P., VAlfonsi F., Tonelli P. (2016). Surgical treatment of oroantral communications. J. Craniofac. Surg..

[12] Puglisi S., Privitera S., Maiolino L., Serra A., Garotta M., Blandino G., Speciale A. (2011). Bacteriological findings and antimicrobial resistance in odontogenic and non-odontogenic chronic maxillary sinusitis. J. Med. Microbiol..

[13] Borogonovo A.E., Berardinelli V.F., Favale M., Maiorana C. (2012). Surgical options in oroantral fistula treatment. Open. Dent. J..

[14] Abuabara A., Cortez L.V., Passeri L.A., De Moraes M., Moreira R.W.F. (2006). Evaluation of different treatments of oroantralqoronasal communications: Experience of 112 cases. Int. J. Oral. Maxillofac. Surg..

[15] Al-Belasy F.A. (2004). The relationship of “shisha” (water pipe) smoking to postextraction dry socket. J. Oral. Maxillofac. Surg..

[16] Balaji S.M. (2008). Tobacco smoking and surgical healing of oral tissues: A review. Indian J. Dent. Res..

[17] Mayfield L., Soderholm G., Hallstrom H., Kullendorff B., Edwardsson S., Bratthall G., Brägger U., Attström R. (1998). Guided tissue regeneration for the treatment of intraosseous defects using a bioabsorbable membrane: A controlled clinical study. J. Clin. Periodontol..

[18] Sørensen L.T. (2012). Wound healing and infection in surgery. The clinical impact of smoking and smoking cessation: A systematic review and meta-analysis. Arch. Surg..

[19] Sørensen L.T., Karlsmark T., Gottrup F. (2003). Abstinence from smoking reduces incisional wound infection: A randomized controlled trail. Ann. Surg..

[20] Krueger K.J., Rohrich R.J. (2001). Clearing the smoke: The scientific rationale for tobacco abstention with plastic surgery. Plast. Reconstr. Surg..

[21] Rudmik L., Mace J.C., Smith M.D. (2011). Smoking and endoscopic sinus surgery: Does smoking volume contribute to clinical outcome?. Int. Forum. Allergy. Rhinol..

[22] Briggs R.D., Wright S.T., Cordes S., Calhoun K.H. (2004). Smoking in chronic rhinosinusitis: A predictor of poor long-term outcome after endoscopic sinus surgery. Laryngoscope.

[23] Twito D., Sade P. (2014). The effect of cigarette smoking habits on the outcome of dental implant treatment. Peerj.

[24] Hurwitz E.E., Simon M., Vinta S.R., Abouleish A.E., Zehm C.F., Shabot S.M., Minhajuddin A. (2017). Adding examples to the ASA-physical status classification improves correct assignment to patients. Anesthesiology.

[25] Mehra P., Jeong D. (2009). Maxillary sinusitis of odontogenic origin. Curr. Allergy Asthma Rep..

[26] Rosenfeld R.M., Piccirillo J.F., Chandrasekhar S.S., Brook I., Kumar K.A., Kramper M., Orlandi R.R., Palmer J.N., Patel Z.M., Peters A. (2015). Clinical practice guideline (update): Adult sinusitis. Otolaryngol. Head Neck Surg..

[27] Vargas-Schaffer G. (2010). Is the WHO analgesic ladder still valid? Twenty-four years of experience. Can. Fam. Physician..

[28] Miloro M., Ghali G.E., Larsen P.E., Waite P.D. (2011). Peterson’s Principles of Oral and Maxillofacial Surgery.

[29] Visscher S.H., Room M.R.F., Sluiter W.J., Von Minnen B., Bos R.R. (2011). Retrospective study on the treatment outcome of surgical closure of oroantral communications. J. Oral. Maxillofac. Surg..

[30] Johnson G.K., Hill M. (2004). Cigarette smoking and the periodontal patient. J. Periodontol..

[31] Chrcanovic B.R., Albrektsson T., Wennerberg A. (2015). Smoking and dental implants: A systematic review and meta-analysis. J. Dent..

[32] Keenan J.R., Veitz-Keenan A. (2016). The impact of smoking on failure rates, postoperative infection and marginal bone loss of dental implants. J. Evid. Based. Dent..

[33] Moraschini V., Barboza E.D. (2016). Success of dental implants in smokers and nonsmokers: A systematic review and meta-analysis. Int. J. Oral. Maxillofac. Surg..

[34] Isola G., Alibrandi A., Rapisarda E., Matarese G., Williams R.C., Leonardi R. (2020). Association of Vitamin d in patients with periodontitis: A cross-sectional study. J. Periodontal Res..

[35] Isola G., Matarese G., Ramaglia L., Pedullà E., Rapisarda E., Iorio-Siciliano V. (2019). Association between periodontitis and glycosylated haemoglobin before diabetes onset: A cross-sectional study. Clin. Oral. Investig..

[36] Jain M.K., Ramesh C., Sanker K., Lokesh B.K.T. (2012). Pedicled buccal fat pad in the management of oroantral fistula: A clinical study of 15 cases. Int. J. Oral. Maxillofac. Surg..

[37] Horowitz G., Koren I., Carmel N.N., Balaban S., Abu-Ghanem S., Fliss D.M., Reiser V. (2016). One stage combined endoscopic and per-oral buccal fat pad approach for large oroantral fistula closure with secondary chronic maxillary sinusitis. Eur. Arch. Otorhinolaryngol..

[38] Gazal G. (2020). Management of an emergency tooth extraction in diabetic patients on the dental chair. Saudi. Dent. J..

